# The dynamics of loss and hope in a world of wars and climate change

**DOI:** 10.3389/fpsyg.2026.1750080

**Published:** 2026-04-07

**Authors:** Johannes Lehtonen, Anna Lehtonen

**Affiliations:** 1 Faculty of Health Sciences, Institute of Clinical Medicine, Psychiatry, University of Eastern Finland, Kuopio, Finland; 2 Finnish Institute for Educational Research, University of Jyväskylä, Jyväskylä, Finland

**Keywords:** Bion, climate change, eco-anxiety, safety prior, societal denial, Winnicott

## Abstract

The environmental and political problems caused by climate change are well known and widely debated, whereas the psychological fears arising from the eco-crisis have been less openly discussed. However, a wealth of empirical data indicates a high prevalence of anxiety and the loss of future perspectives, particularly among young people. The relevant psychological aspects involved are poorly understood. Dealing with eco-related fears is important, as the motivation to work for change depends on the psychological maturity with which multiple challenges of the eco-crisis are faced. The key idea of this study is to propose that the deep-seated fears triggered by the eco-crisis resemble the developmentally early, non-verbal anxieties of human infants. This analogy may help explain why eco-anxiety is difficult to comprehend. Addressing the resistant affects related to eco-crisis may facilitate the acceptance of early, unconscious components of anxiety, thereby reducing its psychological burden. Psychoanalytical perspectives derived from the works of Donald Winnicott, Wilfred Bion, and Sigmund Freud are presented to demonstrate how a psychoanalytical approach can be used to confront their resistance to accepting a crisis and the fears associated with it. The concept of a safe prior was introduced to illuminate the real and developmental nature of the early sense of security, which forms the cornerstone for maintaining psychological safety and cognitive functioning. A brief clinical vignette is presented to highlight how impingement on psychological safety in early infancy can be followed by resistant paranoid fears in adult life, particularly when more mature methods of mastering the experiences of loss are lacking. At the end, the study highlights the sense of omnipotence reflected in the denial exhibited by political leaders who refuse to submit to reality testing in the face of current environmental challenges.

## Introduction

The dynamics of loss and hope touch everyone, especially in a world of wars, biodiversity loss, and climate change. Although news of disasters is a part of everyday life, the experience of loss and hope, of absence and presence, is not a popular subject for discussion. A psychological approach to global problems is often perceived as guilt-provoking as well as devaluing the real nature of the dangers. Avoidance is a natural response when a flood of reports creates psychological uncertainty and requires transcending the habitual boundaries of thinking. Wars and the climate crisis cause at least a sense of existential threat, which includes the fear of losing control of life and its continuation. On the horizon of a familiar and peaceful worldview, the fear of the loss of a meaningful life has emerged.

The gloomy *Weltanschauung* among younger generations has been supported by empirical studies documenting the symptoms of climate anxiety. Empirical studies on the psychological effects of climate change have revealed a high prevalence of anxiety, fear, and sadness among young people. In a worldwide review, 75% of respondents viewed the future as frightening, 67% experienced sadness and fear, 62% reported anxiety, and over half of the participants believed that humanity is doomed (Hickman, 2024).

However, the definition of eco-anxiety differs between studies, and the creation of a taxonomy of climate emotions has been deemed necessary. The term describes difficult emotions and mental states arising from environmental conditions and from knowledge about them (Pihkala, 2018; Pihkala, 2022). Fears can be both tangible and intangible, and more difficult to manage. A distinction is made between climate grief and climate anxiety, as well as between feelings of loss that are threatening and those that are a natural part of growing up to live with the changing environment (maturational loss). Eco-distress can also lead to adversarial development and behavior, instead of internalized symptoms (Pihkala, 2024).

Moreover, eco-anxiety should not be regarded as a psychopathological diagnosis but rather as a healthy response to real environmental dangers that need to be validated and shared and not bypassed (Pihkala, 2024; Bianchi et al., 2022). Eco-anxiety and personal psychopathology can become intertwined, and the latter can enhance the feeling of the former. In adolescence, the most acute forms of eco-anxiety can be serious and connected with suicidal tendencies. Eco-anxiety is often viewed through adult eyes (Hickman, 2024).

## The unconscious nature of the anxiety related to climate change

Given the extensive empirical studies, it is important to realize that conscious affects and objective signs related to change are easier to monitor and evaluate, whereas the threats that affect the unconscious experience of the self and identity are more difficult to master. The aim of this study is to examine the role of unconscious affects that may have a significant but hidden effect on responses to the sustainability of our planet.

The unconscious self is a wide and not precisely defined phenomenological concept. It is primarily approached here from the viewpoint of priorities and future expectations based on previously acquired life experiences and stored in unconscious procedural, episodic, and affective memory (Damasio, 2003). The capacity to symbolize is a limited function of the human mind, and changing the accustomed internalized safety priorities and their organization in the mind is not possible without resistance. Climate change is intertwined with all aspects of living and being human; therefore, it is also a wicked problem in a psychological context.

The embodied affective background tone of consciousness, formed during early development of the mind, persists as a template for adult emotional life (Damasio, 2003; LeDoux, 1996). It represents an undifferentiated awareness of being alive, similar to a primordial form of consciousness that exists within the unconscious layers of the mind. In neuroscience, it is often denoted as “background emotions,” which are both embodied and protomental, and experienced without a connection to verbal consciousness.

In cognitive science, mental experiences are predominantly addressed from sensory-affective and motor perspectives. Primary mental experiences are characterized as embodied, extended, enacted, and evolutionary in a constant interplay between the subject and environment, and they can be understood as a sensorimotor incorporation of the interaction with the environment (Mojica and Di Paolo, 2025). However, the impact of the invisible, homeostatic change in the air we breathe cannot be understood as a sensorimotor experience, as the slow, invisible change in the atmosphere cannot be seen, touched, or smelled. Only large, sudden, and surprising effects, as well as gradually evolving, real, and slow consequences, can be perceived and quantified.

The actual expectations and fears surrounding environmental change, ultimately, remain a *subjective, idiosyncratic experience* that cannot be sharply defined (Pihkala, 2018). These fears are doomed to remain and dwell at the edges of our awareness, if not altogether outside the conscious experience. This situation calls for alternative ways to conceive the reality of the undifferentiated affects that form the core of the sense of security, as well as the actual threat to its disruption when signs of impending danger are perceived.

## The fear of breakdown

Acceptance of unwanted facts, such as climate change, calls for an understanding of their meaning in a relevant context. Without a framework that gives psychological meaning to the facts, the latter remain isolated, their meaning blurred, and attempts at change become invalidated. Our premise is that psychoanalytic insights can widen our perspective on eco-fears, not by explaining them, but by opening hidden psychological agendas.

Ecological fears involve an uneasy, creeping feeling of something overwhelming and unsolvable, reminiscent of separation fears early in the life cycle. The functioning environment is as necessary for human life as early care, food, and security for a suckling infant. In its vague nature, the primordial features of eco-anxiety have corollaries with the early separation anxiety of a child in the absence of a safe adult. As Ogden (1992), p. 183 has put it: “The infant’s relationship to the environmental mother is like the adult’s relationship to air. We take the air we breathe for granted. If we are threatened to be deprived of it, we become acutely and terrifyingly aware of the way in which we are utterly dependent upon it for our lives.” Although early infantile fears are difficult to grasp., attempts to understand their potential contribution to eco-anxiety may illuminate the nature of the disruption in psychological security and be useful in identifying more adaptive ways to use anxiety as a change-promoting force.

A paradox, however, is that uncertainty about gaining and losing a connection, surviving and being left out, is part of the natural life of a newborn. Survival anxiety is part of the evolutionary history of the human mind. It represents a danger that has previously been perceived but was successfully passed with ongoing development. A grasp of such archaic, embodied memories can aid in understanding similar impasses later in life and help to differentiate what is real and serious about the current fears and what represents distressing but vague and unconscious memories from the past that do not have meaning in today’s reality as they did in their own time.

Only indirect means can help orient us to the reality of the undefined flow of primary emotions that are recognized as embodied affects outside verbal consciousness (Pihkala, 2018). The pediatrician and psychoanalyst Winnicott (1974) presented an original clinical viewpoint to understanding the nature of early affective life and the anxieties connected with it. In particular, his clinical description of the threat of psychological breakdown, given in his paper *The Fear of Breakdown*, offers a novel and unusual perspective on how to understand inconceivable anxieties that are subdued by intense repression.

According to Winnicott (1977), a child’s early development rests upon two factors: structural maturation and a facilitating environment. The concern of infant care is not limited to hedonic pleasure. Responding to hunger and other pressing survival needs that matter is like a bridge that carries the child over the birth gap. Winnicott (1977) characterizes the child’s affective life in its early stages as psychosomatic indwelling and psychosomatic collusion. The core of such an early sense of being primarily involves a feeling of being alive. At the heart of it is a sustaining sense of continuity, which also involves the beginning of the experience of time.

If the early sense of continuity remains insecure and is not crystallized into an enduring sense of being connected, psychological threats later in life may revive early embodied anxiety. If the threat is compelling, as in psychotic or severely depressed states, anxiety can be life-endangering (Pao, 1977). Winnicott identified the symptoms of a risk of such a breakdown as a loss of integration, a sense of continuous falling without attachment, the loss of a tie to bodily experience, the loss of a sense of reality, and a failure to form relationships with others.

However, Winnicott paradoxically notes that the underlying fear of breakdown is unfounded because it has already occurred. Fear is a universal experience from the earliest times of individual development, which has not become conscious in its early, nascent stage. It is similar to a premonition of a previously experienced danger that has not been psychologically contained due to the immaturity of the child. It cannot be a part of the child’s contained self and therefore tends to re-emerge when inconceivable threats emerge later in life.

Although his thinking may seem intuitive, the life-sustaining meaning of encounters between the baby and the mother provides a perspective from which to understand what Winnicott meant by the fear of breakdown. Without being attuned to and contained by others, disasters, loss of connections, and perishing threaten children. The changes following birth and adaptation to the new conditions of postnatal life thus provide a natural model for the impending danger that has been experienced in reality but successfully passed through for the continuation of development and a living sense of being. When activated by signs of danger, fear of breakdown tends to prevail in the unconscious unless its transient developmental nature is recognized.

## Bion’s understanding of the preservation of the capacity to think under threat

While Winnicott provides a dynamic scenario for the early experience of sustaining life, Bion (1984) emphasizes the importance of the capacity to maintain thinking, which he calls the alpha function. When faced with overwhelming threats, such as those encountered on the front line in war or in serious forms of the present-day eco-crisis, both the body and the capacity to think are threatened. While Winnicott focuses on the fear of breakdown, Bion’s contribution lies in recognizing the limits of thinking capacity and symbolization, which can prevent a person from drowning in formless paralysis when a perspective for the alternation between hope and despair is not in sight.

Brown (2012) suggests that Bion’s sensitivity to the connection between psychological security and the capacity to think was influenced by his experience in World War I. He served on the front line with the armored troops and had witnessed up close the reality of the destruction of life. Bion mastered the psychological pressure of the battlefield and was able to maintain his capacity for thinking and control of his mind. At the front, he was unyielding and was later awarded the Victoria Cross (Lopez-Corvo, 2003). He had experienced the threat in himself, and his ability to maintain inner security under severe threat to his life made his psychological survival possible. The experience allowed him to understand the fundamental connection that exists between the sense of security and the capacity to think.

Thinking connects elements from the outside and from within and organizes the raw memory imprints (beta elements) into chains that allow the alpha function to develop. When connections arise, chains are formed within a semantic framework that considers facts and allows a capacity to think to emerge. Bion (1984) referred to early childhood development as a natural background for his theory. The genuine bond between the child and reality, represented by the caretaker, arises earlier than cognitive differentiation. The readiness to make cognitive distinctions, such as the difference between external reality and the self, and to endure emotional tensions without immediately releasing them, develops more slowly. Connections arise first, and along with them the budding capacity for thinking; only then can the psychological meaning of the connections emerge.

Bion’s understanding of the meaning of the alpha function and the capacity for thinking can offer relevant insights in a climate context. The ability to tolerate uncertainty and continue searching for an understanding of highly complex data on climate change calls for endurance and challenges the comfort of thinking. Tolerating the discomfort of unwelcome news is easier when the capacity for thought is attuned to the flow of news that lacks definite meaning but only conveys signs of unwelcome development that require evaluation. It is like listening to a quiet but persistent voice of reason, as Freud expressed it, or maintaining radical hope in the midst of facing loss and death as part of an environmental crisis (Pihkala, 2018).

## ‘Fort-da’—the tension of maintaining a sense of connection

In addition to Winnicott and Bion, another original example of how shapeless ambiguity between loss and hope can turn into an initial form of understanding comes from Freud’s (1920) story of his grandson’s ‘fort-da’ game with a wooden reel on a string. The game gives form to the tension between absence and return and the child’s autonomous attempt to manage the anxiety of being left alone.

Freud observed his 1.5-year-old grandson while his mother was away and his father was at the front. The boy used to throw toys on the floor to the point of annoyance and wait for others to pick them up. He then came up with the idea of using a wooden reel with a piece of string attached as a yo-yo, which he threw out of his cot and then pulled back. Each time he threw the reel, he shouted “o-o-o-o” for a long time, and when he pulled the reel back into view, he shouted ‘da.’ The boy’s vocabulary was limited. The long “o-o-o-o” meant ‘fort,’ and ‘da’ meant ‘there.’

The grandfather interpreted the game as an attempt to control the distress caused by his mother’s absence by first throwing the reel away and then pulling it back. This game was like an early version of children’s hide-and-seek games, with joyful rediscovery, gradually giving the child a psychological means to gain control over the separation from and return of his mother. The child had the courage to think about what the loss meant and had a desire to take control of the situation, thereby opening a way out of total dependence.

Freud’s grandson’s game with a wooden reel is a good example of Bion’s theory of the instinct to know, which he denoted by the symbol K and its opposite, –K (Bion, 1984). In the climate context, Bion’s symbolization of and against knowing and not-knowing is reminiscent of the ambivalence that can prevail in believing in or doubting evidence of climate change. Present-day political agendas to stop monitoring climate change indicators suggest that the desire for ignorance may be more powerful than that for knowledge.

## Disruption of life conditions is part of normal development

As Winnicott notes, a threat to the experience of continuity is part of everyone’s life experience. However, due to their ill-defined and non-verbal nature, issues related to early experience are mainly dealt with only on a general level, leaving their real meaning speculative and fuzzy. Therefore, a brief recap of the physiological effects of birth may be called for to emphasize how real and profound the changes are that a child goes through when moving from inside the mother to the reality common to all people (Bradford, 1998).

The child’s life inside the mother is a time of peace, even though the fetal period exposes the child to various sensations, such as those derived from movements and sounds in and outside, as well as the smell and taste of amniotic fluid. In utero, the child does not experience interruptions in physiological homeostasis that would suddenly change its safety. Life in the womb is part of the physiological development within the mother’s body and depends on her health. Psychological security, protecting against external dangers, is not part of fetal life, as the fetus does not face external challenges.

Birth fundamentally alters a child’s physiology and changes the conditions of the child in a fundamental way. An interface is created between the child and the outside world. The alertness level reaches full wakefulness, and the child begins to orient toward the external world. Hormone concentrations and neurochemical transmitter levels increase. All basic vital functions adapt to the external environment. Breathing starts, and oxygenated circulation is directed through the lungs. Fluid balance, digestion, bacterial flora, and the immune system must, for the first time, adapt to the demands of the external world. Sensory organs begin to transmit images from the external world. With the birth cry, the child begins to breathe and actively suck for nourishment from the mother. Sucking requires physiological work, which promotes the child’s capacity for external interaction (Dowling, 1977; Lappi et al., 2007). The child’s entire body is tuned in a new direction at birth when encountering the previously unknown external world.

## The safety prior

The evolving sense of being alive implies a constant fluctuation in the child’s sense of security for physiological reasons. The cycle of hunger, satisfaction, sleep, and wakefulness is repeated several times a day. From this, a regular circadian rhythm is built that is completed and regulated by the mother’s nourishing and calming contributions.

Winnicott (1949) denotes the safe continuation of experience as a ‘going on being’ experience that forms a dynamic, time-bound safety platform for development (Weil, 1970; Pacella, 1980). Recent brain studies have emphasized the importance of continuity in related terms by underscoring the role of the predictive capacity of the mind to anticipate the future by forming predictive priors based on earlier experiences. Priors are activated by predictive coding in order to make sense of the affordances of the external world. According to this principle (also known as Bayesian inference), belief in the prediction of future perception is guided by prior knowledge. In the neurosciences, predictive coding plays a central role in understanding how perception triggers mental expectations and images (Picard and Friston, 2014; Seth, 2021).

A secure sense of continuity, the Winnicottian ‘going on being,’ is like a safety prior of its own kind that resides in the child’s expectations as a default mode of experiencing. It supports confidence in the continuity of experience because it has regularly occurred before. The repeated experience of being fed when hungry and other pressing needs being responded to makes it natural to expect its continuation when new needs occur.

When trust in this experience is established, it gradually turns into a permanent safety prior. Repeated care has, under experimental conditions, been shown to strengthen memory imprints in the brain and help offspring endure separation, pain, and stress (Meaney and Szyf, 2005; Packheiser et al., 2024). The effects of nourishing care have also been confirmed by neurophysiological observations that have demonstrated a direct impact of breastfeeding on the infant’s brain activity, as well as the differential impact of the mother’s voice on the brain function of the child compared to the voice of an alien person (Lehtonen et al., 1998; Lehtonen et al., 2002; Lehtonen et al., 2016; Purhonen et al., 2005).

In summary, postnatal development and the establishment of an internalized safety prior in the infant highlight the vital role of the core feeling of security in the continuation of successful development. Serious impingements on the safety of the self, therefore, tend to trigger primitive modes of anxiety, analogous to the intangible and existential forms of anxiety prompted by the imminent signs of the environmental crisis (Pihkala, 2018; Pihkala, 2022).

Next, a clinical example is provided to illustrate the long-lasting psychological effects of separation trauma in early infancy.

## A clinical vignette

A man with a long work history as a teacher contacted me when his second period of psychotherapy ended because of his therapist’s retirement. He had a history of psychotic breakdown as a young adult following a prolonged period of addictive behavior in different forms. His first therapy helped him resume academic studies and enter a professional life, forming a stable relationship and starting a family.

We met regularly for 16 years, twice a month. The patient was unwilling to undergo frequent sessions. Psychosis was characterized by paranoid beliefs regarding the ill will of his first girlfriend, whom he believed to have influenced his daily life by causing various types of nuisance. I listened to his paranoid beliefs and sometimes questioned them but allowed him to continue, and he was happy for the opportunity to tell me and did not require me to share the beliefs.

We were able to reconstruct, with my active help, his early life story. He was the elder of the two siblings, and his mother gave birth to his sister when he was one year old. The mother had severe complications and was hospitalized for two months, during which time he was completely separated from her. The impact of this early trauma had not been discussed in his previous therapies. The infantile fear of separation had not been worked through but had remained as encapsulated trauma, a permanent void in his basic trust in human relationships.

With my active efforts, separation trauma became the central theme for understanding the background to his paranoid beliefs. It was difficult for him to accept such a connection; however, he slowly admitted that the early separation had been traumatic and had injured his early, infantile safety. There were no words for the trauma; however, its reconstruction was possible, and he was on occasion even able to play with words about the trauma, like a distant effort at working through it. When our relationship ended due to my retirement, his paranoid beliefs were exacerbated, and he received psychopharmacological and electroconvulsive therapy. He had to downshift his life; however, for the first time, we had a meeting during which he no longer mentioned his paranoid beliefs, contrary to what he had obsessively always done before.

His story illustrates the severe, permanent, and resistant-to-change nature of early imprints of disrupted safety and how difficult the later, clinical working through of an encapsulated fear of breakdown can be, much resembling the impasse-like resistance to overcoming the deep fears triggered by severe eco-crises.

## The societal dimension

Climate change has taken several decades to penetrate human consciousness. Opposition to its signs and widespread denial prevailed until the early 2000s. The consistent work on the extensive research data of the Intergovernmental Panel on Climate Change has gradually made it possible to accept climate change as a fact (Flannery, 2005). In addition, the motivation to work toward reducing carbon emissions has also arisen. However, the phenomenon itself, global warming, has been continuously progressing.

Climate change is a strong political issue, and it is reflected in the pressures in countries whose leaders are among the deniers of climate change. The intensity of denial has perhaps even strengthened in recent years, almost as a desperate last attempt to prevent the danger from being perceived as real, as the facts increasingly prove otherwise. How can the denial be understood?

Decades ago, when the environmental change became apparent, it was not difficult to understand that the threat scenarios created a strong need to be incredulous and question the danger signs. The climate is similar to the womb around us, or the mother’s lap in which we live. The disruption of such basic security threatens both physical life and the experiencing mind. It is more natural than strange that there has been a desire to deny obvious signs of change in the environment.

The threat of environmental change evokes a fear of the collapse of ecosystems, resembling the psychological fear of breakdown described by Winnicott (1974). At the heart of the problem is man’s dependence on nature, which is obvious on the one hand but questions the ideal of a free and a scientifically and technologically superior human being in relation to nature. The autonomous and self-contained identity of modern man is seriously questioned in the midst of advanced technological science and industry.

Climate change is a paradox, as it has arisen as a result of technological development and cheap energy, and progress in these areas has been excellent. Humans have truly dominated nature; however, they have also produced a new, uncontrolled problem, the recognition of which does not fit the image of an all-powerful human. The belief in complete independence from nature is an illusion, like a child’s unconscious belief in miracles, which parents should allow the child to foster within reasonable limits without violating the child’s self-esteem (Lehtonen, 1994; Välimäki and Lehtonen, 2009; Lehtonen and Välimäki, 2013).

Disillusionment inevitably follows illusions, and its consequences are not always constructive. Leuzinger-Bohleber (2026) has described the severe impact of disillusionment on maintaining human values and creativity, including the instinct to care for the next generation, which she views as severely threatened in the contemporary cultural climate. When deceived in her confidence in love and deprived of future expectations, Medea turns against her children and, consequently, her own sexual creativity. Leuzinger-Bohleber draws an analogy to this in the loss of trust and courage to have children by today’s young generation due to disillusionment and the uncertainty of a secure future, like an adversarial outcome of the failing mastery of climate anxiety, as described by Pihkala (2024).

The dynamics of loss and hope have an infinite number of faces that vary according to age, perspective, and social status. Coping with issues of the magnitude of climate change is not possible solely at the individual level. Facing these threats requires shared societal efforts, and for this aim, real, not idealized, leadership is required (Välimäki and Lehtonen, 2009; Weintrobe, 2013). Denial of the actions of today’s political leaders is a real problem because it prevents open discussion of the problems and uncertainties of climate change. While denial prevails, the opposite of knowledge, the desire not to know, the “K” by Bion (1984), dominates societal reality.

Weintrobe (2013) has pointed out that primitive anxiety leads to the loss of proportionate, sensible thinking and may lead to an attack on the further development of the human capacity to think, while the real aim of the work is to promote adaptation, which should be in making the anxiety tolerable so that constructive thinking can go on. The limited capacity to symbolize and master complex phenomena, such as the climate knot, works in the same way as denial. Within the complex and ambiguous reality that is difficult to fathom, the meaning of lurking threats easily remains unrecognized.

Omnipotence and actual limits to the capacity to think are like the two sides of the same coin. What may appear to be a lack of care or apathy does not only manifest a lack of concern but rather a lack of the means to express it (Pihkala, 2018). The psychological effects of climate change have created a kind of environmental neurosis in modern man, as its effects question the continuing satisfaction of urgent vital demands for life but simultaneously evoke a strong pressure to repress open discussion of them, mainly because of the lack of means to handle the power of the conflict (Lehtonen and Välimäki, 2013).

Thus, the impact of an environmental crisis is a psychologically wicked problem tied to essential bonds with the societal self-image of modern man. We cannot step out of the environment because we are an intrinsic part of it. Our self-image is deeply rooted in the essential conditions of life, all the way from infancy to adulthood. Changing habits of consumption and other essentials for life inevitably challenge the identity and self-image of men.

Here, however, a specific point of view is the window through which the use of psychoanalytic expertise can become meaningful in facilitating change. Accepting the need to change the self-ideal with its multiple unconscious determinants can never be easy or go without loss and mental pain. The mourning of change can be supported by allowing one of the psychoanalytically informed approaches to work on the dilemma. The circle closes, and we return to the general dynamics of life, which involves working for satisfaction and facing problems, failures, and successes, as in Freud’s grandson’s game with the mother surrogate, who was here, present, and soon absent again. This was an alternation of security and fear, which the grandson knew how to handle using a wooden rail to approach basic uncertainty.

Leaders committed to denial do not want to participate in this game. They are not ready to explore and test the presence or absence of the world’s life-sustaining conditions, and they do not dare to play with dangerous alternatives in their imagination and think about how things could be if they were not the way they want them to be. Things must be as the denier wants. If not, things must be changed as desired, as far as this can be done. The denier’s strong belief in omnipotence cannot be changed by presenting arguments.

However, as long as the work of adaptation primarily concentrates on threats and anxieties, the blurring of perspective easily occurs. The final aim is not only to adapt to the malaise produced by changes but also to strive for a better and healthier environment. Even if only reality can teach us how to live with the facts of presence and absence, successful environmental and psychological work with climate change is aimed at leading to a brighter and healthier future and not only to submission and loss (Bianchi et al., 2022). This may be difficult to understand as long as the climate debate focuses more or less solely on the deprivation that is deemed to follow the acceptance of change. After all, nature and a healthy ecosystem are precious homes of life and real sources of hope, which should be respected and cared for.

This is a matter for everyone and not only for political leaders. It especially challenges today’s educators to guide younger generations not to run away, but rather to investigate the true state of affairs and look for new, creative, and hope-giving solutions.

## Data Availability

The original contributions presented in the study are included in the article/supplementary material, further inquiries can be directed to the corresponding author.
